# The impact of premature birth on auditory-visual processes in very preterm schoolchildren

**DOI:** 10.1038/s41539-024-00257-3

**Published:** 2024-07-06

**Authors:** Marion Décaillet, Solange Denervaud, Cléo Huguenin-Virchaux, Laureline Besuchet, Céline J. Fischer Fumeaux, Micah M. Murray, Juliane Schneider

**Affiliations:** 1 Department of Radiology, Lausanne University Hospital and University of Lausanne, Lausanne, Switzerland; 2 The Sense Innovation and Research Center, Lausanne and Sion, Lausanne, Switzerland; 3 Clinic of Neonatology, Department of Mother-Woman-Child, Lausanne University Hospital and University of Lausanne, Lausanne, Switzerland

**Keywords:** Human behaviour, Education

## Abstract

Interactions between stimuli from different sensory modalities and their integration are central to daily life, contributing to improved perception. Being born prematurely and the subsequent hospitalization can have an impact not only on sensory processes, but also on the manner in which information from different senses is combined—i.e., multisensory processes. Very preterm (VPT) children (<32 weeks gestational age) present impaired multisensory processes in early childhood persisting at least through the age of five. However, it remains largely unknown whether and how these consequences persist into later childhood. Here, we evaluated the integrity of auditory-visual multisensory processes in VPT schoolchildren. VPT children (*N* = 28; aged 8–10 years) received a standardized cognitive assessment and performed a simple detection task at their routine follow-up appointment. The simple detection task involved pressing a button as quickly as possible upon presentation of an auditory, visual, or simultaneous audio-visual stimulus. Compared to full-term (FT) children (*N* = 23; aged 6–11 years), reaction times of VPT children were generally slower and more variable, regardless of sensory modality. Nonetheless, both groups exhibited multisensory facilitation on mean reaction times and inter-quartile ranges. There was no evidence that standardized cognitive or clinical measures correlated with multisensory gains of VPT children. However, while gains in FT children exceeded predictions based on probability summation and thus forcibly invoked integrative processes, this was not the case for VPT children. Our findings provide evidence of atypical multisensory profiles in VPT children persisting into school-age. These results could help in targeting supportive interventions for this vulnerable population.

## Introduction

Humans live in a multisensory environment, with information available to the different senses that need to be combined or segregated appropriately to generate coherent perceptions and behavior. Examples of the perceptual and behavioral benefits of multisensory processes extend from stimulus detection^1–4^ to speech perception^5,6^, food appreciation^7,8^, as well as body consciousness^9,10^. Multisensory processes can also enhance decision-making and different types of learning (e.g., both object recognition^11^ and object recognition memory^12^ are enhanced by congruent audiovisual stimuli, and unisensory, visual improvement of motion sensitivity can be improved by the presence of sounds (i.e., visual-motion learning) thanks to auditory stimuli^13^).

In adults, multisensory processes have been well investigated^14–16^. By contrast, substantially less is known regarding the developmental trajectory of multisensory processes and how early life experience influences this trajectory. This is precisely the case of prematurity, for which long-term multisensory development has only been slightly investigated, even though it concerns roughly 10% of births worldwide, 10% of those being very preterm births (i.e., births occurring before 32 gestational weeks)^17,18^. Very preterm births may include adverse neurodevelopmental consequences, such as sensory processing and integration. These difficulties are present during early development from the first months through toddlerhood^19–21^. Here, we investigated the consequences of very preterm birth on sensory processing and integration that persist through school age.

Premature infants have immature nervous systems and experience altered sensory environments that could impact their functional development. During their hospitalization, infants are exposed to a completely different ex-utero context than their full-term peers. In the Neonatal Intensive Care Unit (NICU), very preterm infants are surrounded by machines and thus are more exposed to mid- and high-frequency sounds and brighter, unnatural lighting^22^ while receiving less (or atypical) tactile or vestibular stimulation^23^. In addition, whereas during pregnancy rhythmic stimulation with synchronization between the different sensory systems is frequent, this is disrupted during their stay in the NICU^24^. This atypical environment and the necessary medical procedures could lead to various situations of dystimulation (i.e., inappropriate stimulations)^25–27^. Indeed, the stimuli experienced by premature neonates are oftentimes less targeted for infants’ neurodevelopment, instead focusing on their medical wellbeing. One consequence may therefore be less infant-directed and thus more incongruous stimuli^28^, which may in turn not be accurately integrated by the neonate and that could lead to future sensory difficulties such as hypo- and hyper- sensitivity^29^. In support, at discharge, premature infants present diminished brain responses to (multi)sensory stimuli as well as atypical non-linearities when compared to their full-term peers^20^. Moreover, multisensory (but not unisensory) brain responses at hospital discharge were predictive of typical scores on both the Infant/Toddler Sensory Profile (ITSP) at 12 months of age and metrics of internalizing tendencies at 24 months of age^20^. Likewise, differences in unisensory brain responses are also observed, including reduced mean amplitudes of auditory event-related potentials (ERPs) to consonant contrasts in posterior temporal and frontal electrodes^19^, or reduced tactile ERPs at fronto-central electrodes^30^. As they grow up, young preterm children aged 2–5 years old continue to show sensory alterations across all sensory modalities (for a review, see Bröring et al.^26^). However, there is a gap in the literature, as only a few studies have been conducted concerning long-term sensory consequences at school age. Through a sensory questionnaire, Wickremasinghe et al. found that nine-year-old very preterm children have atypical response patterns, including both hypo-responsivity and hyper-responsivity in various senses^31^. Bröring et al. conducted a case-controlled study and found that very preterm schoolchildren aged ~9.2 years present hyposensitivity and hypersensitivity, more specifically, in tactile perception, kinesthesia and graphesthesia^32^. Additionally, using a set-shifting task they also derived a measure of cross-modal interactions and observed no differences in accuracy or reaction times between preterm and full-term children. However, their task might assess cross-modal attention processes with constant updating of the motor response, rather than strictly multisensory integration^33^. Therefore, while this fundamental work sheds light on hyper- or hypo-sensory perception without impacting cross-modal interactions, no research has been conducted without a task that required matching different sensory information, focusing then only on high-level processes.

It is currently known is that prematurely born schoolchildren are more likely to show long-term neurodevelopmental cognitive impairments and neuropsychiatric disorders^34–37^. At school age, 8-year-old very preterm children show poorer executive function skills even though some of them caught up many of their delays by the age of 12^38–41^. Additionally, their processing speed and working memory notably predict their neurological impairments and their school difficulties^42,43^. Indeed, processing speed mediates impairments in executive functions, which in turn play a role in school readiness^44^.

Several works have drawn links between multisensory integration and some behaviors and cognitive abilities. First, atypical multisensory processes in early infancy have been associated with altered sensory profiles at 12 months of age and internalizing tendencies at 24 months of age^20^. Second, in healthy schoolchildren, low-level multisensory abilities predict higher-level cognition such as working memory and fluid intelligence^45^. Auditory-visual integration is also associated with reading abilities at a young age (between 5–8 years), whereas it is not the case afterward, underscoring how auditory-visual integration is important for learning to how to read^46^. Additionally, several correlations between multisensory integration and IQ have been reported. In a large study with 5 to 12-year-old schoolchildren, Birch & Belmont observed a positive association between IQ and multisensory integration that decreased with age^46^. More recently, Barutchu and colleagues have found that multisensory facilitation was not associated with IQ or reading abilities between 6 and 11 years of age in a quiet environment^47–49^. Nevertheless, when multisensory facilitation exceeded probability summation in noise, it was correlated with a higher IQ^48^. Rose et al. examined potential associations between sensory processing abilities at the ages of 7 months and 1 year on IQ scores throughout childhood in full-term and preterm children^50–52^. They found that this relationship between sensory processing and cognitive outcome is present during the entire childhood until 11 years of age, but that it weakened thereafter. To our knowledge, these are the only studies that investigated these associations in preterm children. If indeed these relationships between multisensory processes and cognitive abilities are also valid with very preterm schoolchildren, it would likely help to identify children most at-risk. As altered multisensory processes may be a precursor or a predictor of these cognitive impairments, support could be adapted accordingly.

In schoolchildren, as well as adults, a simple detection task provides an efficient means of quantitatively assessing multisensory processes. This task typically involves a button-press response to the presentation of each stimulus in a randomized sequence of intermixed unisensory (i.e., auditory or visual) and multisensory (i.e., the simultaneous presentation of both auditory and visual stimuli) events, whose timing is unpredictable. From such a task, one can compare accuracy and reaction times to multisensory stimuli versus each unisensory condition to ascertain if performance is more accurate and/or faster under multisensory conditions; a phenomenon often referred to as the redundant signals effect (e.g., ref. ^53^). It can in turn be queried whether multisensory facilitation as measured via the redundant signals effect is explainable by a race model or instead by a co-activation model^53,54^ as well as by the age when these processes become mature^47,55^. When data are explainable by the race model, information from the different senses can independently initiate the motor response, and probability summation can account for any facilitation of behavior. By contrast, co-activation is invoked when facilitation of behavior exceeds probability summation, and by extension information from the different senses must interact prior to initiating the motor response^54^. Such notwithstanding, neural response interactions have been observed both when the race model is violated and satisfied^56,57^. Studies with healthy children have yielded mixed results, showing that multisensory integration and facilitation development is variable and not completely mature during childhood^58^. During the first 6 months of life, multisensory matching^59^ and the transfer of information from one sensory modality to another start to become mature^60,61^. Multisensory facilitation develops through the first months, indeed young infants show multisensory facilitation from at least one month, but only infants aged 8 months and older started to exhibit some evidence for co-activation under certain conditions^62^. During childhood, Barutchu et al. have not observed a clear developmental trajectory of multisensory integration^47^. Whereas there was no clear evidence of co-activation at 6 and 10 years, children aged 7–9 years presented evidence of violation of the race model. Therefore, multisensory facilitation seems to be still immature and fluctuating even in late childhood. Similarly, Brandwein et al. have also found that even if a multisensory facilitation effect appeared at a young age, its processes became mature around adolescence^55^. More specifically, the extent of co-activation, measured as the magnitude as well as number of children exhibiting such, increased with age. One postulation is that this facilitation process is not automatic and effortless for children, but rather may rely on the maturation of high-level cognitive and top-down processes^47^. To our knowledge, however, none of these prior studies specifically reported or accounted for the gestational age at birth of their participants.

Thus, in view of previous research and the lack of long-term information, the primary aim of the current study was to characterize the multisensory processing profile of very preterm children at school age. Using a simple detection task without the need for stimulus associations, low-level stimulus processing could be investigated. Our secondary aim followed from evidence that multisensory gains in simple detection (i.e., the percentage difference in mean reaction time between the multisensory and the faster unisensory stimulus) can predict global cognition in schoolchildren^45^. Therefore, we assessed correlations between multisensory gains observed in very preterm schoolchildren and neuropsychological test scores. Except for one ongoing study by Neel et al. investigating the benefits of a multisensory intervention in the neonatal intensive care units, there is no proven sensory support intervention^63^. Accurately understanding the specificity of sensory difficulties in very preterm children would enable us to tailor support and to create appropriate sensory rehabilitation for neonates and children at an early age.

## Results

### Perinatal characteristics of the VPT children

Several perinatal factors linked to prematurity could be related to later neurodevelopment (for details, see Table 1).

**Table 1:** Perinatal factors of the VPT children

Measures		Mean (s.d.)/number (%)	Range
GA in weeks		27 (2.80)	25–31
Birth weight (grams)		906 (227)	610–1590
Apgar 5 min		7 (1)	3–9
Ventilation (hours)			
	Non-invasive	1120 (406)	490–2131
	Invasive	89 (132)	0–534
Post-natal steroids (number)		4 (14.29%)	
Intraventricular hemorrhage (number)			
	Grade I	5 (17.86%)	
	Grade II	3 (10.71%)	
	Grade III	1 (3.57%)	
	Grade IV	2 (7.14%)	
Bronchopulmonary dysplasia (number)			
	Mild	6 (21.43%)	
	Moderate	5 (17.86%)	
	Severe	4 (14.29%)	
Hospitalization (days)		83 (22)	51–121

### Demographics

While sex and age were not different between VPT and FT children, FT children had a significantly higher socio-economic status. VPT children scored in the normal range for processing speed and working memory index on the WISC. In addition, their IQ evaluated with the WISC was also in the norm (for details, see Table 2).

**Table 2:** Demographic data

Measure	VPT	FT	*p*-value	Cohen’s d
*N* (% girls)	28 (53.57%)	23 (43.48%)	0.48	NA
Age (chronological) in yrs	8.80 (0.51)	9.00 (1.65)	0.584	0.162
SES	57.2 (24.8)	78.2 (18.7)	0.004	1.02
WISC- Total (IQ)	105 (13.3)	NA	NA	NA
WISC – Processing speed	99.4 (15.8)	NA	NA	NA
WISC – Working Memory	102 (17.2)	NA	NA	NA
Fluid Intelligence	53(14.2)	84.5(11.3)	<0.001	2.45

Mean ± SD are indicated in parentheses, except for the percentage of girls. Regarding the SES, note that data from 5 individuals were missing for the FT group and from 3 individuals for the VPT group. The norms for the WISC scores are 100 ± 15.

### Auditory and visual acuity of the VPT children

During the neonatal period, auditory evoked potentials were classified as normal for all VPT infants. And by the age of 8, none of them had any hearing impairment. Regarding vision, two neonates suffered from grade I or II retinopathy of prematurity. One was treated with laser and the second one normalized without treatment. Both had no persistent sequalae. At 8 years old, 10 children were wearing corrective glasses for various pathologies, including 9 with complete correction and 1 without binocular vision. Moreover, one VPT child suffered from strabismus and was undergoing neuro-visual rehabilitation.

### Simple detection task

#### Sensory dominance

Sensory dominance, operationally defined here as the modality resulting in faster unisensory RTs in each participant (see also^64^ and^65^ for similar approaches), was similar between VPT and FT children (χ^2^ = 0.48, *p* = 0.49). There was a significantly greater proportion of visual dominance compared to auditory dominance in both FT children (78% vs. 22%, *p* = 0.011) and VPT children (86% vs. 14%, *p* < 0.001).

#### Stimuli dominance

Regarding the visual stimuli, cloud and star stimuli yielded similar reaction times in VPT children (M_cloud_ = 528, SD_cloud_ = 71 vs M_star_ = 531 SD_star_ = 99.9, *t*_*Student*_(54) = -0.16, *p* = 0.88, *Cohen’s d* = −0.04) and also in FT children (M_cloud_ = 370, SD_cloud_ = 39.9 vs M_star_ = 370 SD_star_ = 37.6, *t*_*Welch*_(43.8) = 0.04, *p* = 0.97, *Cohen’s d* = 0.01).

Regarding the auditory stimuli, whistle and yawn stimuli yielded similar reaction times in VPT children (M_whistle_ = 593, SD_whistle_ = 115 vs M_yawn_ = 617 SD_yawn_ = 126, *t*_*Welch*_(53.6) = −0.73, *p* = 0.47, *Cohen’s d* = −0.19) and FT children (M_whistle_ = 389, SD_whistle_ = 47.7 vs M_yawn_ = 400 SD_yawn_ = 49.7, *t*_*Student*_(44) = −0.80, *p* = 0.43, *Cohen’s d* = −0.24).

#### Accuracy

The Friedman test on mean Accuracy showed a main effect of Stimulus Condition, (χ^2^(2) = 7.32, *p* = .0.03), which was due to lower accuracy for auditory (*M* = 91.6%, *SD* = 8.32) than for multisensory (*M* = 94.7%, *SD* = 7.92) stimuli (i.e., Dubin–Conover pairwise comparison: *p* = 0.007). But visual (*M* = 92.1%, *SD* = 11.1) presented an intermediate accuracy which was not significatively different from auditory and multisensory conditions.

The Kruskall–Wallis test showed that the general accuracy of FT children (*M* = 92.3%, *SD* = 8.45) was similar to the accuracy of VPT children (*M* = 93.2%, *SD* = 6.69), (χ^2^(2) = 0.01, *p* = 0.94), as well as for each condition separately (see Table 3). The inclusion of SES and the Fluid Intelligence as covariates did not change this pattern of results.

**Table 3:** Accuracy

Stimulus Condition	VPT	FT	Chi-square	*p*	ε^2^
Visual	91.6% (12.6)	92.7% (9.38)	0.003	0.953	6.83e-5
Auditory	92.5% (8.11)	90.4% (8.61)	0.77	0.38	0.02
Multisensory	95.4% (7.06)	93.8% (8.94)	0.24	0.63	0.004

Accuracy as a percentage (SD indicated) for each modality on the simple detection task and its comparison between VPT and FT groups.

#### Reaction times

The Friedman test on mean RTs showed a main effect of Stimulus Condition, (χ^2^(2) = 74.9, *p* < 0.001), which was due to generally faster RTs for multisensory (*M* = 415 ms, *SD* = 92.8) than for either visual (*M* = 457 ms, *SD* = 103) or auditory (*M* = 510 ms, *SD* = 138) stimuli (all Durbin–Conover pairwise comparisons between each stimulus condition *p* < 0.001).

**Fig. 1:**
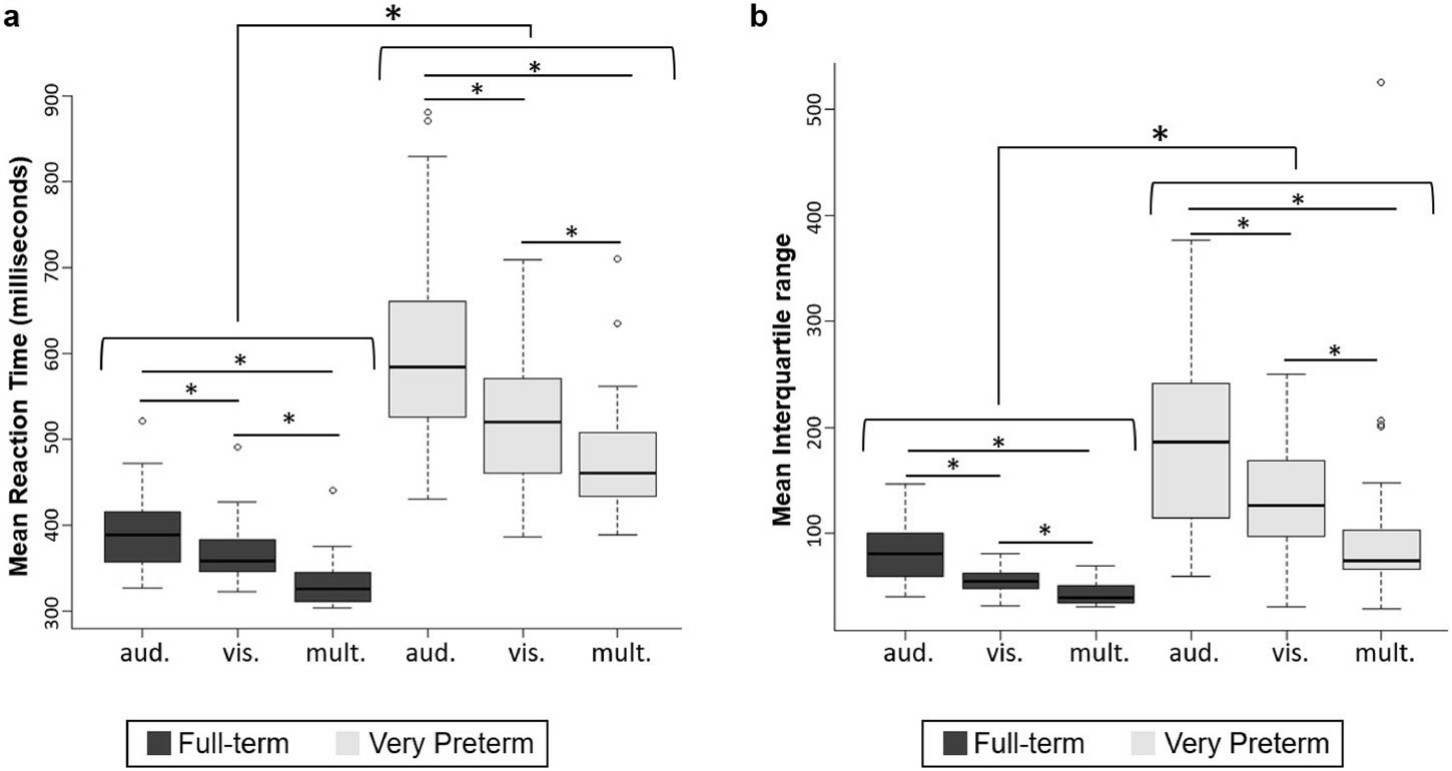
Distribution of reaction times and interquartile range for both groups as a function of stimulus modality. **a** Mean reaction times for each stimulus condition separated by group. **b** Mean interquartile range for each stimulus condition separated by group. Aud. auditory, vis. visual, mult. multisensory, *p < 0.001, center line = median, bounds of the box = 25 and 75 percentiles, whisker = maximum and minimum observation within 1.5 times of interquartile range, circle (“o”) = outliers.

The Kruskall–Wallis test showed a main effect of Group; FT children (*M* = 366 ms, *SD* = 37.9) being generally faster than VPT children (*M* = 538 ms, *SD* = 79.1), χ^2^(1) = 35.3, *p* < 0.001, ε^2^ = 0.71 (Fig. 1a). This was the case for each stimulus condition (i.e., visual: *M*_*FT*_ = 370 ms, *SD* = 38.2 vs *M*_*VPT*_ = 529 ms, *SD* = 80.7, χ^2^(1) = 33.8, *p* < 0.001, ε^2^ = 0.68; auditory: *M*_*FT*_ = 395 ms, *SD* = 47.8 vs *M*_*VPT*_ = 605 ms, *SD* = 113, χ^2^(1) = 34.7, *p* < 0.001, ε^2^ = 0.69; and multisensory: *M*_*FT*_ = 335 ms, *SD* = 31.6 vs *M*_*VPT*_ = 481 ms, *SD* = 79.1, χ^2^(1) = 35.3, *p* < 0.001, ε^2^ = 0.71). This pattern of results remained the same when adding the SES and the Fluid Intelligence as covariates.

#### Variability

The Friedman test on IQRs showed a main effect of Stimulus Condition, (χ^2^(2) = 56.7, *p* < 0.001), which was due to smaller IQR for multisensory (*M* = 76.8, *SD* = 76.8) than for either visual (*M* = 97.9, *SD* = 58.4) or for auditory (*M* = 140, *SD* = 82.7) stimuli; the latter of which also significantly differed from each other (all Durbin–Conover pairwise comparisons between each stimulus condition *p* < 0.001).

The Kruskall–Wallis test showed a main effect of Group; FT children (*M* = 60.7 *SD* = 11.3) having generally smaller IQR than VPT children (*M* = 141, *SD* = 60.8), χ^2^(1) = 33.6, *p* < 0.001, ε^2^ = 0.67 (Fig. 1b). This was the case for each stimulus condition (i.e., visual: *M*_*FT*_ = 55.8, *SD* = 12.3 vs *M*_*VPT*_ = 133, *SD* = 58.7, χ^2^(1) = 25.5, *p* < 0.001, ε^2^ = 0.51; auditory: *M*_*FT*_ = 83.4, *SD* = 30.6 vs *M*_*VPT*_ = 186, *SD* = 83.5, χ^2^(1) = 21.4, *p* < 0.001, ε^2^ = 0.43; and multisensory: *M*_*FT*_ = 43.0, *SD* = 9.78 vs *M*_*VPT*_ = 105, *SD* = 95.2, χ^2^(1) = 21.8, *p* < 0.001, ε^2^ = 0.44).

#### Multisensory gain

##### Reaction times

The percentage multisensory gain on RT was similar in FT children (*M* = 9.08%, *SD* = 2.29) and in VPT children (*M* = 7.52%, *SD* = 7.87), (*t*(32.4) = 1, *p* = 0.33; Cohen’s d = 0.27).

##### Variability

The percentage multisensory gain on IQR was similar in FT (*M* = 16.6%, *SD* = 9.61) and in VPT children (*M* = 9.61%, *SD* = 48.7), (*t*(38.6) = 0.69, *p* = 0.50; Cohen’s d = 0.19).

#### Race model inequality

**Fig. 2:**
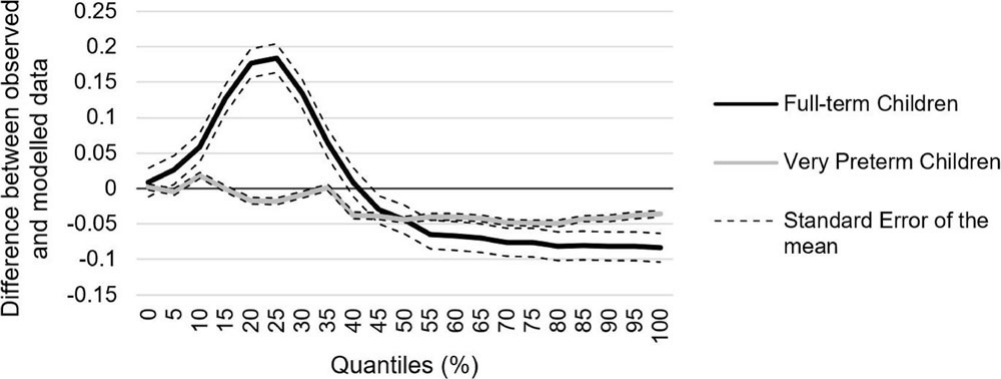
Race model inequality. Very preterm and full-term children’s cumulative probability distributions for the auditory, visual, simultaneous (i.e., audio-visual) stimuli and for the values predicted by the race model.

There was slight violation of the Race Model inequality in VPT children for the 0, 10, 15, 25, 30, and 35 quantiles (Fig. 2). However, in no case was the cumulative probability for the multisensory condition significantly higher than the cumulative probability for the values predicted by the race model (for details, see Table 4). By contrast, the race model was significantly violated in FT children for the 0, 5, 10, 15, 20, 25, 30, 35, and 40 quantiles. The cumulative probability for the multisensory condition was significantly higher than the cumulative probability for the values predicted by the race model for these eight quantiles (for details, see Table 4).

**Table 4:** Simultaneous vs predicted cumulative probability

	VPT				FT			
	Cumulative Probability				Cumulative Probability			
Quantile (%)	Simultaneous	Predicted	*t*	*p* value	Simultaneous	Predicted	*t*	*p* value
0	0.03 (0.03)	0.03 (0.03)	0.00	0.5	0.01 (0.01)	0.002 (0.01)	2.87	0.004**
5	0.06 (0.09)	0.07 (0.06)	−0.41	0.66	0.03 (0.03)	0.003 (0.01)	4.00	<0.001***
10	0.15 (0.20)	0.14 (0.13)	0.77	0.22	0.09 (0.08)	0.03 (0.06)	4.35	<0.001***
15	0.22 (0.24)	0.21 (0.20)	0.33	0.37	0.22 (0.16)	0.09 (0.13)	5.69	<0.001***
20	0.31 (0.27)	0.31 (0.27)	−0.01	0.50	0.38 (0.22)	0.19 (0.19)	7.49	<0.001***
25	0.44 (0.30)	0.43 (0.29)	0.79	0.22	0.53 (0.23)	0.33 (0.23)	6.88	<0.001***
30	0.56 (0.28)	0.56 (0.30)	0.14	0.45	0.65 (0.20)	0.50 (0.24)	7.11	<0.001***
35	0.57 (0.28)	0.64 (0.30)	1.31	0.10	0.73 (0.19)	0.65 (0.22)	3.93	<0.001***
40	0.74 (0.19)	0.75 (0.18)	−0.43	0.66	0.79 (0.17)	0.76 (0.19)	1.20	0.12
45	0.81 (0.15)	0.84 (0.12)	−1.53	0.93	0.82 (0.17)	0.83 (0.18)	−0.52	0.69
50	0.86 (0.12)	0.89 (0.08)	−1.85	0.96	0.84 (0.17)	0.87 (0.15)	−1.39	0.91
55	0.88 (0.10)	0.92 (0.07)	−2.11	0.98	0.85 (0.17)	0.9 (0.015)	−2.43	0.99
60	0.90 (0.10)	0.94 (0.05)	−2.41	0.99	0.87 (0.17)	0.92 (0.14)	−2.72	0.99
65	0.91 (0.10)	0.95 (0.04)	−2.87	1	0.88 (0.17)	0.93 (0.13)	−2.86	1
70	0.92 (0.09)	0.96 (0.04)	−3.16	1	0.88 (0.17)	0.93 (0.13)	−3.22	1
75	0.93 (0.08)	0.98 (0.03)	−3.08	1	0.89 (0.17)	0.94 (0.12)	−3.30	1
80	0.93 (0.08)	0.98 (0.03)	−3.15	1	0.89 (0.17)	0.94 (0.11)	−3.34	1
85	0.94 (0.08)	0.98 (0.03)	−3.00	1	0.89 (0.17)	0.95 (0.11)	−3.15	1
90	0.94 (0.07)	0.98 (0.02)	−3.29	1	0.89 (0.17)	0.95 (0.11)	−3.15	1
95	0.95 (0.07)	0.99 (0.02)	−3.09	1	0.89 (0.17)	0.95 (0.11)	−3.16	1
100	0.96 (0.07)	0.99 (0.02)	−3.27	1	0.90 (0.17)	0.96 (0.10)	−3.03	1

The average cumulative probability (and s.d.) for the simultaneous condition with that predicted by the race model as well as the corresponding paired *t*-test (one-sided) and *p*-values for all quantiles in VPT children (left) and FT children (right). *p < 0.05, **p < 0.01, ***p < 0.001

#### Relationships between multisensory gains and neuropsychological scores in VPT

VPT children scored in the normal range on all selected scales of the SPM questionnaire and WISC-V tests (for details, see Table 5). By controlling for gestational age and SES, partial Spearman correlations were carried out. The multisensory gain was not significantly correlated with any of the clinical or cognitive measures (for details, see Table 6).

**Table 5:** Neuropsychological tests for VPT children

Neuropsychological Tests	Mean (s.d.)	Norms	IQR
SPM – Vision	48.9 (6.80)	<60	13
SPM – Audition	48.8 (8.85)	<60	13
SPM – Total	51.3 (7.11)	<60	6
WISC – Processing Speed	99.4 (15.8)	100 (15)	16
WISC – Working Memory	102 (17.2)	100 (15)	18
WISC - Total	105 (13.3)	100 (15)	15

Mean = T-scores.

*SPM* Sensory Processing Measure, *WISC* Wechsler Intelligence Scale Children.

**Table 6:** Correlations between multisensory gain and clinical and cognitive measures

Variables	Multisensory Gain	*p* value
SPM – Vision	−0.24	0.32
SPM – Audition	−0.05	0.86
SPM – Total	0.11	0.66
WISC – Working Memory	0.02	0.92
WISC – Processing Speed	−0.02	0.94
WISC – IQ Total	−0.15	0.51
Digit Span	0.12	0.59
Digit Span - Ascending	0.06	0.78

Partial correlations controlling for SES and gestational age.

## Discussion

Despite their importance in daily life and their contributions to cognitive functions, how multisensory processes develop has been under-investigated, especially in children more at risk for neurodevelopmental and neuropsychiatric disorders, such as VPT children. Here, we provide some of the first data on low-level multisensory processes in VPT children at school age. While neuropsychological assessments were in the normal range, RTs on the simple detection task were generally both slower and more variable in VPT schoolchildren. VPT children did exhibit multisensory facilitation like their FT peers. However, for VPT children, this facilitation was fully explained by mechanisms based on probability summation. By contrast, multisensory facilitation in FT children exceeded predictions based on probability summation and instead entailed integrative processes (see also Barutchu et al., for similar findings in FT children^66^). These results suggest that both sensory as well as multisensory processes are altered in a long-lasting way by early-life events.

First, our findings reveal that VPT children showed generally slower RT to unisensory and multisensory stimulus conditions. This was the case even though both FT and VPT children exhibited similar, near-ceiling accuracy rates for all stimulus conditions, suggesting that this difference was not the result of a speed-accuracy trade-off or general inattentiveness (see ref. ^67^ for discussion). Likewise, IQ scores from VPT children were within the normal range, implying that the observed slowing was not the result of a general difference in global cognition, in particular the processing speed. It thus appears that their sensory detection capacity (i.e., accuracy) is not impaired, but it takes them longer to respond to stimulation. Accuracy and RT in the FT children here are highly concordant with values reported by prior works^55,66^. The slowing of RTs in VPT children is similar to what was observed in 6-year-old VPT by de Kieviet et al. in visual attention task^68^ (see also Aarnoudse-Moens et al. about processing speed^69^). Our results thus indicate that this slowing persists into later childhood. In contrast, when it concerns high-level detection (i.e., sensory matching task), VPT children have not exhibited slower RT^32^. Therefore, while they present impaired low-level multisensory detection mechanisms (i.e., simple detection task), VPT children might rely on other – perhaps compensatory - mechanisms when completing higher-level cognitive tasks. Whether and how such invocation of compensatory strategies and mechanisms contribute to the observed higher risk for learning differences and poorer scholastic achievement in VPT individuals remains to be fully characterized, particularly if such derive, even partially, from the persistent general slowing we observed here.

Second, despite more variable simple RT across all sensory modalities, VPT children nonetheless exhibited percentage gains equivalent to those of their FT peers both when mean RT and their IQR were quantified. This pattern of results runs somewhat counter to what might otherwise be predicted from a strict application of the so-called principle of inverse effectiveness^70^. That is, it could be contended that the slower and more variable RT observed in VPT children are indicative of reduced stimulus effectiveness (compared to FT children). As such, it would follow that larger percentage gains would be expected in the VPT children. However, it is also the case that the RT distributions in response to unisensory conditions in the VPT children were less overlapping. Prior work has shown that larger gains are observed when these distributions are more superimposed^71^ (see also Otto et al.^72^). Such notwithstanding, we consider it particularly noteworthy that performance variability was significantly decreased (i.e., became less variable) under multisensory stimulus conditions in both full-term and preterm schoolchildren alike and despite general differences in RT speed. Multisensory stimulus contexts may thus provide an access point for remediating situations where highly variable behavior may be a contributor to poorer performance and cognition, as has been reported, for example, in children with ADHD^73^.

Third, while multisensory gains in FT children necessitated integrative processes, this was not the case here for our group of VPT children. We would hasten to note that even though there was no violation of probability summation in VPT children, this does not necessarily exclude the presence of nonlinear neuronal response interactions^56,74^. Such notwithstanding, the presence of multisensory gains in VPT children that are moreover fully explained by probability summation suggests different underlying neural processes subserving multisensory processes across the two groups of children. Therefore, further investigations are needed to comprehensively understand those mechanisms using brain mapping and/or imaging tools. As these multisensory and integrative processes are thought to mature during late childhood in FT children^55,66^, an additional question arises as to whether differences we observe in VPT reflect a delay or a persistent deficit. Accordingly, future studies should include adolescents and adults to investigate the complete developmental trajectory of multisensory processes in preterm-born individuals.

Multisensory integration has been found to have a cascading effect on higher-order cognitive skills (e.g., Denervaud et al.^45^; for a review see ref. ^75^). Therefore, this atypical sensory profile could not only affect performance in laboratory settings, but also daily life activities, including schooling and academic performance. At school, audition and vision are often paired and contribute to various learning processes, in particular regarding language and reading. Indeed, poorer readers exhibit impaired audiovisual integration^46,76,77^. In addition, multisensory integration also interacts with memory functions^12,78^. One possibility is that altered multisensory processes in VPT schoolchildren could explain in part their academic difficulties in many domains^79–81^. It is therefore particularly important to develop sensory intervention and rehabilitation strategies to enhance multisensory integration as early as possible. Several protective measures are already being implemented to reduce the impact of the hospitalization in the NICU and related atypical sensory stimulation. Indeed, since the last 20 years, implementation of the developmental care has spread out and developed, by encompassing the broader concept of family integrated care^82^. This care approach regroups different neuroprotective measures such as skin-to-skin contact, aiming at minimizing stress and pain, and partnering with families to create the best healing environment as possible^83^ according to the recommendations of the European Foundation for the Care of Newborn Infants (EFCNI) and the World Health Organization (WHO). Another resource developed is the single-family room, which allows for lower noise levels, and provides better light control, often with more natural light. But these rooms also come with other disadvantages and cannot be adapted to all centers^84^. In 2011–2012, at the time of their neonatal hospitalization, the VPT children of our cohort had beneficiated from some of these strategies, but the protective measures have since improved and therefore represent a promising way of preventive actions of long-term multisensory impairments that need to be consolidated.

Finally, contrarily to FT children where the multisensory gain predicted measures of global cognition^45^, in VPT children cognitive and clinical sensory measures did not correlate with percentages of multisensory gain. This result combined with the fact that they only showed little high-level cognitive deficit could indicate the use of compensatory mechanisms, akin to what has been invoked in other works that did not observe performance differences between VPT and FT children. Concerning the Sensory Processing Measure, this questionnaire might assess the sensory reactivity (i.e., behavioral response to a sensory stimulus), whereas the simple detection task relies more on the sensory sensitivity (i.e., perception of the sensory stimulus). These two concepts might be independent of each other^85^. It is likewise possible that the failure to observe significant correlations here may be due (at least in part) to the rather homogenous and high scores on the neuropsychological tests.

It is noteworthy that our findings are in line with atypical patterns of multisensory processes found among various neurodevelopmental disorders. Indeed, while there is a lack of studies regarding prematurity, multisensory integration has been well-examined in diverse neurodevelopmental disorders. Using the same type of simple detection task, Harrar et al. have found the same pattern of results (i.e., RT slowing in every stimulus conditions and fewer race model violations) with adults with developmental dyslexia^76^. Likewise, Salles et al., have also observed slower RT and diminished percentage multisensory gains in patients with Prader-Willi syndrome^86^, which itself has a higher incidence among preterm than full-term births^87^. In children, atypical multisensory integration has been found in individuals with autism^88^ and ADHD^89^. However, most of these studies, including those with healthy children, do not report gestational age, but it is likely important that researchers systematically report such information.

Some limitations of this study are worth mentioning. One limitation is that the VPT group is generally healthier than what is commonly reported in the literature^90,91^. Although this situation is hopefully becoming increasingly common thanks to advances in healthcare and their global dissemination/access, it does not represent the entire population of VPT children. Therefore, future research might want to investigate multisensory processes in VPT children with more serious impairments as these are likely children particularly at risk for long-term sequelae. Another consideration is the difference in SES between the groups. While we included SES as a covariate in our analyses here, this difference nonetheless underscores the importance of considering such factors both in research and in applications of findings when ideating interventions and/or rehabilitation strategies^92^. Moreover, fluid intelligence was not assessed with the same measure in both groups here, which may partially explain the difference between VPT and FT children. Nonetheless, the pattern of our results did not change when it was added as covariate, reinforcing the robustness of our findings. Another potential consideration is that the VPT children were carrying out the task as part as their clinical follow-up, which theoretically could have induced more stress than for FT children. We should also mention that the limited pre-existing FT children cohort prevented us from carrying out pairwise matching. For all these latter limitations, future studies should include FT control children matched in the same setting (i.e., context, tests) as the VPT children to ensure the reproducibility of our findings. In the current study, we focused on visual and auditory sensory modalities, but as young VPT children show impairments in all sensory modalities^26^, future studies should test other senses especially those which are more mature at full-term birth (e.g., tactile^93^ and vestibular processes^94^), as well as behavioral responses, to evaluate more comprehensively their multisensory profiles. In this regard, it would likewise be informative for future works to obtain scholastic achievement data to allow for a more direct comparison between sensory and multisensory response profiles and everyday skills.

In conclusion, these findings highlighted the persistent effects of prematurity into school age that manifest as altered sensory and multisensory processing. Therefore, there is a need for sensory interventions in the neonatal period such as proposed by Neel et al.^63^ to improve the standard developmental care in the NICU as well as in early childhood to enhance their unisensory and multisensory processes that provide critical scaffolding for cognitive abilities and behavior throughout the lifespan^4^. Indeed, some supportive therapies are offered such as occupational therapy for sensorial hypersensitivity, visual therapy by orthoptists or hearing aids. But these therapies are only proposed to children with overt impairments and are not intended for cognitive purposes. Thus, our results provide evidence for developing new supportive interventions strengthening multisensory cognitive processes and targeting all vulnerable children.

## Methods

### Participants

All parents provided their written, informed consent for their child to participate. The project was approved by the state ethics committee (Commission cantonale d'éthique de la recherche sur l'être humain (CER-VD), protocol no. 2019-01056) and conforms to principles outlaid in the 2013 Declaration of Helsinki.

#### Very preterm children

This is an observational study of fifty-one very preterm (VPT) children previously hospitalized in a level III NICU who were recruited in the main project entitled Long-term impact of early nutritional and pain management in very preterm infants on brain health and function at their birth^95^. From October 2019 to October 2021, on their last follow-up appointment at eight years old, 41 children participated (i.e., ten children did not take part in the study because of death (*n* = 2), lost contact at a previous follow-up visit (*n* = 2), complex social situation (*n* = 2), unreachable parents (*n* = 2) or refusal to participate (*n* = 2)). Finally, due to incomplete testing, the final sample included 28 very preterm children (M_gestational age_ = 27 weeks, SD = 2.80), aged 8–10 years chronological (M_age_ = 8.80, SD = 0.51, 15 girls).

#### Full-term children

As a reference group, data from twenty-three full-term (FT) children aged 6-11 (M_age_ = 9.00, SD = 1.65, 11 girls) were selected from an existing database based on age (*t*(25.4) = 0.55, *p* = 0.584) and gender (χ^2^(1) = 0.515, *p* = 0.48). Due to a limited number of participants, we could not do pair-wise matching. They participated in a parallel study about neurodevelopment and schooling experience and underwent the same simple detection task detailed below. Their non-prematurity was assessed through a questionnaire, but no precise gestational age (GA) was collected.

### Tasks and procedure

The testing of the VPT children took place during the follow-up appointment at the Development Unit of the Lausanne University Hospital following their premature birth. They were tested individually in a quiet room. FT children were tested individually either in a quiet room at Lausanne University Hospital or at their school. The task was presented and controlled using the E-Prime 2.0 Software (Psychology Software Tools, Pittsburgh, PA) displayed on a PC laptop. It was assessed by neuroscience researchers.

#### Simple detection task

**Fig. 3:**
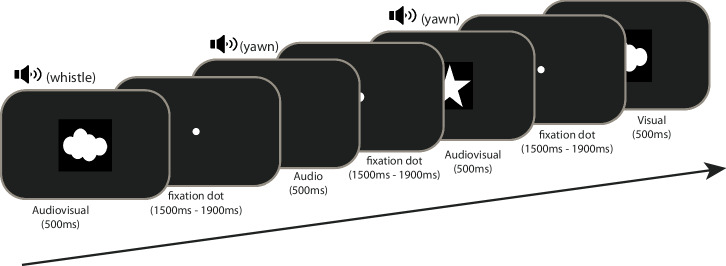
Task design. Illustration of the different types of stimuli and sequence of events.

Visual (V), auditory (A) or audiovisual (AV) stimuli were presented to children. The visual stimuli were composed of a white cloud, or a white star presented in the middle of a black background. The auditory stimuli were either of two types of tones (44,100 Hz digitization; 16 bits stereo) that varied in terms of their spectral composition: a whistle ranging from 18,700 Hz to 19,600 Hz and a yawn ranging from 20 Hz to 21,000 Hz. The audio-visual stimuli were the synchronous presentation of the visual and auditory stimuli. Each type of stimulus was randomly presented 20 times. Therefore, the children completed 60 trials. Stimulus duration was 500 ms and during the pseudo-randomized inter-stimulus interval (i.e., 1500, 1600, 1700, 1800 and 1900 ms), a small white fixation dot was presented (for an illustration of the task, see Fig. 3). Children were asked to press the keyboard spacebar as fast as possible each time they saw or heard something. We recorded both accuracy and reaction time.

#### Socioeconomic status (SES)

Given the differences between experimental versus clinical contexts, SES was not assessed with the same questionnaire for the VPT and FT groups. SES of the VPT group was calculated with the Largo score, with values ranging from 1 to 6^96^. Lower scores correspond to higher education degrees (i.e., university education), and higher scores correspond to shorter formal education (i.e., elementary school, occupations without apprenticeship or professional designation). We summed and averaged the scores of both parents, converted them to a percentage and then subtracted them from 100% to allow for comparison with SES scores from the FT group. SES of the FT group was assessed according to both parents’ educational level and current job, both scoring from 1 to 4 (1 representing lower level)^97^. We also summed and averaged scores across both parents and then converted them to a percentage to allow for comparison between groups.

#### Clinical batteries

During their follow-up appointment, several clinical batteries and questionnaires were also completed by VPT children or their parents. Clinical batteries were assessed by two clinical psychologists. Clinical batteries were not completed by the FT children.

##### Sensory processing measure (SPM)

This instrument consists of a questionnaire completed by the parents and that assesses diverse sensory processing, praxis, and social participation^98^. Parents rated each of the 75 statements about their child’s behavior by whether it had occurred never/sometimes/often/always in the past month. In the present study, the scales *Vision*, *Audition*, as well as the total score were considered.

##### Wechsler intelligence scale for children (WISC-V)

This tool evaluates the intellectual ability of children^99^. In this study, we considered the total IQ score, the *Working Memory Index* and *Processing Speed Index*. The working memory index is measured using two subtests, the *Picture Span* (i.e., remembrance of a sequence of pictures in the correct order and the selection of these images from a larger picture array) and the *Digit Span* (i.e., the recall of a sequence of numbers in the original, reverse, and ascending order). Processing speed is composed of the subtest *Code* (i.e., transcription of a digit-symbol code using a specific key) and the subtest *Symbol Search* (i.e., detection of a target within a line of symbols).

##### Fluid intelligence

To evaluate the fluid intelligence, VPT children performed the *Matrix Reasoning* subtest from the WISC-V. Children had to select the missing piece that completed a sequence of colored visual pattern matrices. The full-term children completed a similar task. They were assessed with the black and white version of Raven’s Progressive Matrices^100^ in which they were asked to choose the missing piece from a matrix of black-and-white visual patterns. The two scores were then converted in success rate to be comparable.

### Data analyses

For reaction time (RT) analyses from the simple detection task, only responses given in a valid reaction time range (i.e., RTs ranging between 150 ms and 2000 ms, and RT ± 3 *SD* for an individual child collapsed across all conditions) were included^45,101^. Across all the participants, ~6.93% of the trials were discarded. Accuracy was computed as the percentage of detected stimuli (i.e., response given within the valid reaction time, corresponding to non-discarded trials). In addition, we computed the individual Interquartile Range (IQR) of the RTs distribution for each condition. Analyses were performed using Jamovi (Version 0.9) Computer Software and R.3.6.3^102^. The statistical significance level was set at *p* ≤ 0.05.

First, we assessed and compared the frequency of sensory dominance (i.e., the unisensory modality with faster RTs) between VPT and FT children with a Chi-squared test. Then, we compared the unisensory and multisensory accuracy, RT, and IQRs as well as the multisensory gain between VPT and FT children. As some accuracy, reaction time and IQR distributions were not normally distributed in both groups and because the homogeneity of variance was violated (Levene’s Test *p* < 0.05 for all stimulus conditions), we performed nonparametric tests to investigate the impact of Stimulus Condition (i.e., Friedman Test) and of Group (i.e., Kruskal–Wallis Test) on the accuracy, mean RTs, and IQRs. In addition, a first multisensory gain was calculated as the relative difference between the mean reaction time to multisensory and the faster unisensory condition (i.e., Eq. (1)). A second multisensory gain was computed in the same way but based on IQRs instead of RTs. We performed a *t*-test (two-tailed) on these two multisensory gains to compare both groups.

1 $$\frac{{{\boldsymbol{Mean\; RT}}}_{{\boldsymbol{fastest\; unisensory\; condition}}}\,-\,{{\boldsymbol{RT}}}_{{\boldsymbol{multisensory\; condition}}}}{{{\boldsymbol{RT}}}_{{\boldsymbol{fastest\; unisensory\; condition}}}}\times 100$$

Second, to investigate the underlying multisensory processes and to compare the presence of the ‘redundant signal effect’ (RSE), we computed the Race Model inequality^54^ in both groups. Inaccurate trials where children failed to respond in the given RT period were removed from the analyses. Each subject’s RT for each condition was treated individually. The cumulative probability (CP) distributions were calculated for each condition after having been normalized in terms of the percentile of the range of reaction times across all stimulus conditions (i.e., bin widths of 5% were used in the current study). Then group-averaged cumulative probability values were calculated for each modality. The predicted values by the model were calculated by Eq. (2) and compared to the observed audio-visual cumulative probability for each bin. For each percentile where the predicted values were exceeded by the observed simultaneous values (i.e., audio-visual stimuli), the race model was violated, and the co-activation hypothesis was favored. Paired *t*-tests (one-tailed) were performed to test the reliability of this violation.

2 $$({{\boldsymbol{CP}}}_{{\boldsymbol{auditive}}}+{{\boldsymbol{CP}}}_{{\boldsymbol{visual}}})-({{\boldsymbol{CP}}}_{{\boldsymbol{auditive}}}\times {{\boldsymbol{CP}}}_{{\boldsymbol{visual}}})$$

Finally, to explore if the multisensory gain in very preterm children was related to the neuropsychological measures, partial correlations were performed between clinical and cognitive measures, and the multisensory gain with SES and gestational age as covariables. As *Vision* and *Audition* scales were not normally distributed, partial Spearman correlations were computed.

### Reporting summary

Further information on research design is available in the Nature Research Reporting Summary linked to this article.

### Supplementary information

Reporting Summary

## Data Availability

The anonymized raw data from the simple detection task ask, the demographic data from both groups, and the neuropsychological scores and neonatal characteristics of the very preterm children are publicly available on the Open Science Framework repository (https://osf.io/gnm8r/?view_only=d730b97761684826b20548d4c9870b4c).

## Code availability

The codes that support the findings of this study are available from the corresponding author, Marion Décaillet, upon reasonable request.
