# Supplementary material for: The impact of premature birth on auditory-visual processes in very preterm schoolchildren
Source: NPJ Sci Learn. 2024 Jul 6;9:42. doi: 10.1038/s41539-024-00257-3 (PMC11227572; doi:10.1038/s41539-024-00257-3)
Supplement: Supplementary file 1 — Reporting Summary [file 41539_2024_257_MOESM1_ESM.pdf]

nature portfolio

Corresponding author(s): DAPR - 01026

Last updated by author(s): Jun 3, 2024

Reporting Summary

Nature Portfolio wishes to improve the reproducibility of the work that we publish. This form provides structure for consistency and transparency in reporting. For further information on Nature Portfolio policies, see our Editorial Policies and the Editorial Policy Checklist.

Statistics

For all statistical analyses, confirm that the following items are present in the figure legend, table legend, main text, or Methods section.

n/a	Confirmed	
☐	☒	The exact sample size (*n*) for each experimental group/condition, given as a discrete number and unit of measurement
☐	☒	A statement on whether measurements were taken from distinct samples or whether the same sample was measured repeatedly
☐	☒	The statistical test(s) used AND whether they are one- or two-sided<br>*Only common tests should be described solely by name; describe more complex techniques in the Methods section.*
☐	☒	A description of all covariates tested
☐	☒	A description of any assumptions or corrections, such as tests of normality and adjustment for multiple comparisons
☐	☒	A full description of the statistical parameters including central tendency (e.g. means) or other basic estimates (e.g. regression coefficient) AND variation (e.g. standard deviation) or associated estimates of uncertainty (e.g. confidence intervals)
☐	☒	For null hypothesis testing, the test statistic (e.g. F, t, r) with confidence intervals, effect sizes, degrees of freedom and P value noted<br>*Give P values as exact values whenever suitable.*
☒	☐	For Bayesian analysis, information on the choice of priors and Markov chain Monte Carlo settings
☒	☐	For hierarchical and complex designs, identification of the appropriate level for tests and full reporting of outcomes
☐	☒	Estimates of effect sizes (e.g. Cohen's *d*, Pearson's *r*), indicating how they were calculated

Our web collection on statistics for biologists contains articles on many of the points above.

Software and code

Policy information about availability of computer code

Data collection	All methods are detailed in the manuscript and computer / software code used for data collection are described or information is provided to readers on where such can be obtained.
Data analysis	Analyses have been detailed in the Methods section and include the use of open source software.

For manuscripts utilizing custom algorithms or software that are central to the research but not yet described in published literature, software must be made available to editors and reviewers. We strongly encourage code deposition in a community repository (e.g. GitHub). See the Nature Portfolio guidelines for submitting code & software for further information.

Data

Policy information about availability of data

All manuscripts must include a data availability statement. This statement should provide the following information, where applicable:

- Accession codes, unique identifiers, or web links for publicly available datasets
- A description of any restrictions on data availability
- For clinical datasets or third party data, please ensure that the statement adheres to our policy

The anonymised raw data from the simple detection task ask, the demographic data from both groups, and the neuropsychological scores and neonatal characteristics of the very preterm children are publicly available on the Open Science Framework repository (https://osf.io/gnm8r/?view_only=d730b97761684826b20548d4c9870b4c).

Research involving human participants, their data, or biological material

Policy information about studies with human participants or human data. See also policy information about sex, gender (identity/presentation), and sexual orientation and race, ethnicity and racism.

Reporting on sex and gender	Gender was collected for both groups and results applied for both gender. Gender was assigned according to the medical record. Full-term children from the reference group were selected based on gender. There were 15 girls and 13 boys in the very preterm children group. And 11 girls and 12 boys in the full-term group. Gender was not a prerequisite for inclusion to the study. Likewise, we did not perform any sex-based or gender-based analysis, as the outcome of the present study does not relate to either sex or gender.
Reporting on race, ethnicity, or other socially relevant groupings	We used and reported their socioeconomic status. For the very preterm children, it was assessed according to the educational level of both parents. The parents were asked about their work and their educational level was deduced from this by the child's psychologists. And for the full-term children, it was assessed according to both parents' educational level and current job and calculated with the Largo score. Parents completed a questionnaire about their educational level and current profession.<br>We used it as a covariate in our analyses.
Population characteristics	We used the fluid intelligence and the socioeconomic status as covariate, as well as the gestational age for the very preterm children.
Recruitment	All surviving participants of an existing cohort of very preterm neonates born before 30 gestational weeks between February 2011 and May 2013, who were recruited in the CHUV tertiary care NICU were eligible.
Ethics oversight	La Commission cantonale d'éthique de la recherche sur l'être humain (CER-VD)

Note that full information on the approval of the study protocol must also be provided in the manuscript.

Field-specific reporting

Please select the one below that is the best fit for your research. If you are not sure, read the appropriate sections before making your selection.

☐ Life sciences ☒ Behavioural & social sciences ☐ Ecological, evolutionary & environmental sciences

For a reference copy of the document with all sections, see nature.com/documents/nr-reporting-summary-flat.pdf

Behavioural & social sciences study design

All studies must disclose on these points even when the disclosure is negative.

Study description	Very preterm children are part of a quantitative observational cohort study.
Research sample	28 very preterm children (GA between 25 and 31 weeks), including 15 girls, previously hospitalized in the level III NICU at the Lausanne University Hospital in Switzerland. They were aged between 8 and 10 years. They were generally healthier than what is commonly reported in the literature and although this is increasingly the case nowadays, it does not represent the entire population of very preterm children.<br>23 full-term children in the reference group were aged between 6 and 11 years and included 11 girls.
Sampling strategy	There was no sample size for this specific study as we recruited the very preterm children from the initial longitudinal cohort. Recruitment of previously enrolled participants in the initial cohort has limited the total number of eligible children, and therefore the sample is a convenience from the existing cohort.
Data collection	The very prterm children were tested at their follow-up appointment at the Development Unit of the Lausanne University Hospital. They were tested individually. The simple detection task was presented and controlled using the E-Prime 2.0 Software displayed on a PC laptop. The researcher remained with the participant during the task. The Wechsler Intelligence Scale for Children was assessed by a psychologist and parents completed some questionnaires.<br>Full-term children were tested individually either in a quiet room at Lausanne University Hospital or at their school and researcher also stayed with the participants.
Timing	For very preterm children: October 2019 to October 2021<br>For full-term children: 2018
Data exclusions	No data were excluded for this specific study.<br>Initial exlcusion criteria were parental refusal or inability to seek consent or parents who did not want to be aware of incidental findings.
Non-participation	Ten children did not take part in the study because of death (n=2), lost contact at a previous follow-up visit (n=2), complex social situation (n=2), unreachable parents (n=2) or refusal to participate (n=2). In addition, 3 very preterm children did not perform the simple detection task during the appointment.

Randomization	Allocation was not random but based on their gestational age. We used fluid intelligence and socioeconomic status as covariates.

Reporting for specific materials, systems and methods

We require information from authors about some types of materials, experimental systems and methods used in many studies. Here, indicate whether each material, system or method listed is relevant to your study. If you are not sure if a list item applies to your research, read the appropriate section before selecting a response.

Materials & experimental systems

n/a	Involved in the study
☒	☐ Antibodies
☒	☐ Eukaryotic cell lines
☒	☐ Palaeontology and archaeology
☒	☐ Animals and other organisms
☒	☐ Clinical data
☒	☐ Dual use research of concern
☒	☐ Plants

Methods

n/a	Involved in the study
☒	☐ ChIP-seq
☒	☐ Flow cytometry
☒	☐ MRI-based neuroimaging

Plants

Seed stocks	*Report on the source of all seed stocks or other plant material used. If applicable, state the seed stock centre and catalogue number. If plant specimens were collected from the field, describe the collection location, date and sampling procedures.*
Novel plant genotypes	*Describe the methods by which all novel plant genotypes were produced. This includes those generated by transgenic approaches, gene editing, chemical/radiation-based mutagenesis and hybridization. For transgenic lines, describe the transformation method, the number of independent lines analyzed and the generation upon which experiments were performed. For gene-edited lines, describe the editor used, the endogenous sequence targeted for editing, the targeting guide RNA sequence (if applicable) and how the editor was applied.*
Authentication	*Describe any authentication procedures for each seed stock used or novel genotype generated. Describe any experiments used to assess the effect of a mutation and, where applicable, how potential secondary effects (e.g. second site T-DNA insertions, mosiacism, off-target gene editing) were examined.*
